# Investigation of Motor Learning Effects Using a Hybrid Rehabilitation System Based on Motion Estimation †

**DOI:** 10.3390/s24113496

**Published:** 2024-05-29

**Authors:** Kensuke Takenaka, Keisuke Shima, Koji Shimatani

**Affiliations:** 1 Graduate School of Environment and Information Sciences, Yokohama National University, Yokohama 240-8501, Japan; 2 Faculty of Environment and Information Sciences, Yokohama National University, Yokohama 240-8501, Japan; 3 Faculty of Health and Welfare, Prefectural University of Hiroshima, Mihara 723-0053, Japan; shimatani@pu-hiroshima.ac.jp

**Keywords:** hybrid rehabilitation, motor learning, motion estimation, functional electrical stimulation

## Abstract

Upper-limb paralysis requires extensive rehabilitation to recover functionality for everyday living, and such assistance can be supported with robot technology. Against such a background, we have proposed an electromyography (EMG)-driven hybrid rehabilitation system based on motion estimation using a probabilistic neural network. The system controls a robot and functional electrical stimulation (FES) from movement estimation using EMG signals based on the user’s intention, enabling intuitive learning of joint motion and muscle contraction capacity even for multiple motions. In this study, hybrid and visual-feedback training were conducted with pointing movements involving the non-dominant wrist, and the motor learning effect was examined via quantitative evaluation of accuracy, stability, and smoothness. The results show that hybrid instruction was as effective as visual feedback training in all aspects. Accordingly, passive hybrid instruction using the proposed system can be considered effective in promoting motor learning and rehabilitation for paralysis with inability to perform voluntary movements.

## 1. Introduction

Common examples of rehabilitation for stroke victims towards recovery of upper limb function for everyday living [1] include physical therapy transitive motion exercises, motor imagery-based treatment, and constraint-induced therapy. However, these methods are limited by issues such as therapist scarcity and the difficulty of objective/quantitative assessment and verbal/manual guidance [2,3].

Rehabilitation using robots and functional electrical stimulation (FES) has previously been proposed to address these issues [2]. In robotic rehabilitation, robotic devices are used to apply force to joints, thereby assisting user movement [4,5]. This allows the user to learn precise movements on an ongoing basis without therapist assistance. In this field, Frisoli et al. trained stroke victims using an exoskeleton robot for upper limb motor assistance, producing significantly improved scores in Fugl–Meyer Assessment (FMA), a comprehensive evaluation method for motor dysfunction [6]. Lum et al. compared the effects of robot-assisted movement training with conventional techniques for post-stroke rehabilitation of upper limb motor function, finding that the robot group exhibited greater improvement in the proximal movement portion of Fugl–Meyer assessment than the control group [7]. However, movement training using an exoskeleton-type robot did not improve the user’s capacity for appropriate muscle contraction.

In FES rehabilitation, peripheral nerves are electrically stimulated for appropriate muscle contraction and movement [8]. Chan et al. reported significantly improved FMA upper limb scores in stroke victims after multiple training sessions using FES motor induction [9]. In addition, we previously proposed a human-to-human joint motion transmission system using FES and electromyography (EMG) signals [10] to support rehabilitation with intuitive interaction and teaching of joint motion/muscle contraction via determination of motion states based on EMG signals and provision of FES for motion reproduction. Current challenges with FES include the generation of large amounts of torque to joints and the teaching of precise motion trajectories.

Against this background, hybrid rehabilitation has been another area of research [11]. This method seeks simultaneous capacity for precise joint movement using a robot and FES-based indication of muscle contraction, and has been reported to achieve greater improvement than the stand-alone method [12]. Nam et al. researched detection of motor intent from EMG signals generated by muscle contraction, reporting that joint movement can be controlled and trained using FES, soft actuators, and exoskeleton robotics, thereby significantly improving upper limb motor function in stroke victims [13]. It has similarly been found to promote motor learning through rehabilitation triggered by EMG signals [14,15]. However, control here is based on a predetermined threshold for single-muscle contraction, even though human joints have multiple degrees of freedom and are controlled by the coordinated contraction of various muscles. Accordingly, training for multiple movements based on the contraction of single muscles is problematic under the current approach.

Here, we extend the basic concept of an EMG-driven hybrid rehabilitation system allowing motion estimation proposed in [16]. The system enables accurate evaluation of multiple joint movements based on coordinated muscle contractions, robot control, and FES based on estimated motion for instruction on trajectory and muscular activity. In an experiment, training for specific 2-DOF movement of the wrist joint (pointing) was performed. As a caution, however, it should be noted that the experiment’s sample size was limited and analysis was based only on a single index. Therefore, further examination of the motor learning effect is required.

This paper investigates in detail the training effects on pointing movements using an EMG-driven hybrid rehabilitation system with motion estimation. To quantitatively evaluate the motor learning effect of the proposed system, analysis is conducted on pointing movements performed by the experimental subjects using various indices in terms of accuracy, stability, and smoothness.

This paper details training effects on pointing movements using the EMG-driven hybrid rehabilitation system with motion estimation. Analysis was conducted in terms of accuracy, stability, and smoothness with quantitative evaluation of related motor learning influence. Section 2 describes the system, Section 3 outlines the evaluation, Section 4 covers the results, and Section 5 provides a conclusion and outlines future work.

## 2. Hybrid Rehabilitation System with Motion Estimation [16]

**Figure 1:**
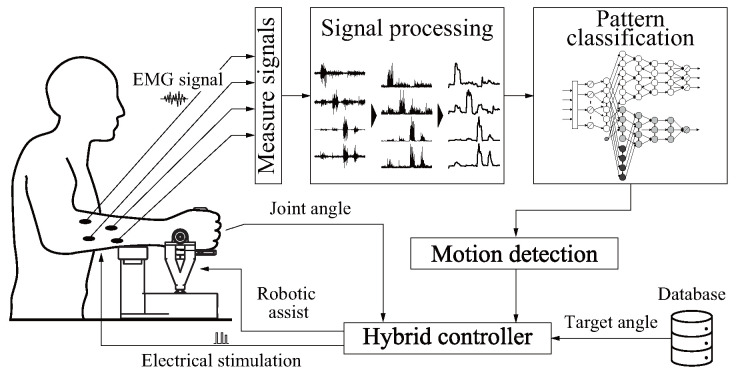
Concept of hybrid rehabilitation system based on [16].

The concept for EMG-driven hybrid rehabilitation is shown in Figure 1. The system evaluates EMG signals from the user’s skin surface, and the results are input to a multi-motion discriminator that evaluates intended movement. Appropriate motion trajectories and contraction levels are instructed by robot assistance and FES, respectively, based on predefined data.

### 2.1. Hardware

**Figure 2:**
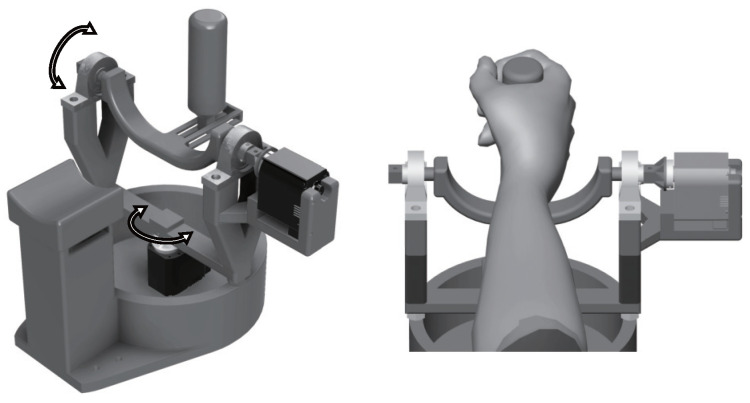
Assistance robot. The arrows in the figure indicate the driving direction of the robot.

Figure 2 illustrates the developed assistance robot. The wrist joint assistance robot is controlled with two compact high-torque servo motors (B3M-SC-1170-A, Kondo Kagaku (Tokyo, Japan); maximum torque: 7.6 Nm; 51 × 32 × 39.5 mm) with serial communication using a USB-connected PC. This enables assistance against strong resistance, such as user spasticity, with the same level of force as that provided by a therapist. The servo-motor range of motion for each axis is ±45 degrees, enabling assistance with 2-DOF wrist joint movement (excluding pronation and supination) and monitoring of joint angles/motor torque during movement.

An external safety switch allows immediate servomotor shutdown. The range of servomotor movement can be set in advance to match the user’s joint capacity.

### 2.2. Software

#### 2.2.1. Signal Processing and Feature Extraction

EMG monitoring and preprocessing are based on [17]. This method is simple, universal, and widely used in machine learning EMG identification [18,19]. EMG signals monitored from *L* pairs of electrodes attached to the subject are A/D-converted with sampling frequency of $f_{s}$ Hz and the effective frequency elements are extracted using a second-order bandpass Butterworth filter (cutoff frequency $f_{c}^{\mathrm{low}}$, $f_{c}^{\mathrm{high}}$ Hz), then full-wave rectification is applied to each signal. Next, $E_{l}\left(t\right)$ is determined via smoothing with a second-order low-pass Butterworth filter (cutoff frequency $f_{c}$ Hz) to extract amplitude information. Here, $E_{l}^{\prime }\left(t\right)$ is a signal normalized using the maximum $E_{l}^{\max}\left(t\right)$ value of each channel when the user performs the desired motion voluntarily in advance, excluding the EMG signal $E_{l}^{\mathrm{st}}\left(t\right)$ monitored at rest from smoothed rectified data for removal of baseline wandering noise, as follows:

(1) $$\begin{array}{c} E_{l}^{\prime }\left(t\right)=\frac{E_{l}\left(t\right)-E_{l}^{\mathrm{st}}\left(t\right)}{E_{l}^{\max}\left(t\right)-E_{l}^{\mathrm{st}}\left(t\right)}. \end{array}$$

The values are then normalized such that the sum of all channels is 1 to prevent a single muscle contraction from skewing the overall feature vector. This allows for independent treatment of muscle coordination patterns and individual contraction levels, and is robust against feature value changes caused by force intensity. The feature vector $x\left(t\right)={\left[x_{1}\left(t\right),x_{2}\left(t\right),\ldots ,x_{l}\left(t\right),\ldots ,x_{L}\left(t\right)\right]}^{T}$ is then defined as

(2) $$\begin{array}{c} x_{l}\left(t\right)=\frac{E_{l}^{\prime }\left(t\right)}{\sum_{l=1}^{L}E_{l}^{\prime }\left(t\right)}. \end{array}$$

The subject’s muscle activation level F(t) is defined as

(3) $$\begin{array}{c} F\left(t\right)=\frac{1}{L}\sum_{l=1}^{L}E_{l}^{\prime }\left(t\right), \end{array}$$

with the presence of the system drives based on the muscle activation level F(t) and a predefined threshold. As human joint movement involves the coordination of multiple muscles, the direction of motion can be estimated relatively accurately from these coordination patterns x(t). System stability is enhanced by determining the drive of the system based on the overall muscle activity level F(t). Probabilistic evaluation of user capacity enables training on multiple movements to be performed via coordinated multiple-muscle contraction.

#### 2.2.2. Motion Estimation

In the proposed system, the user’s muscle contraction is assessed by a probabilistic neural network; only when the user attempts a movement with appropriate muscle contraction is the hybrid system used to instruct the user to perform the movement. This allows the user to be constantly aware of their own movements and to take the initiative in rehabilitation, representing an improvement compared to methods where the system prescribes movements in advance and performs training.

A recurrent probabilistic neural network [20] is used as a discriminator to evaluate the subject’s motion. The network encompasses a hidden Markov model with a Gaussian mixture model, and is capable of accurately discriminating temporally fluctuating EMG signals.

The discriminator is first trained with the muscle contraction pattern x(t) of the target movement, and voluntary muscle contraction is evaluated with input of this pattern to the discriminator after learning. The posterior probability for movements learned from the subject is determined, and the motion with the highest value is taken as the intended one. This enables the determination of multiple movements based on the subject’s intent based on learning of EMG signal changes associated with individual differences in muscle mass and electrode misalignment.

#### 2.2.3. Robot Control

The proposed hybrid system has instruction and training control modes. In instruction mode, motion is determined from EMG signals associated with the subject’s intent and is instructed through a minimum jerk model that reaches a predefined target point in the range of $T_{\mathrm{instruct}}$ s based on the determination. The minimum jerk model generates a motion trajectory such that the integral of the time derivative of acceleration (known as jerk) is minimized from start to finish, and is used as a normative model for natural human motion [21]. The trajectory of motion from the start point $P_{0}$ to the end point $P_{T}$ in time *T* is determined as

(4) $$\begin{array}{c} P\left(\tau \right)=P_{0}+\left(P_{T}-P_{0}\right)\left(6\tau^{5}-15\tau^{4}+10\tau^{3}\right), \end{array}$$

where τ=t/T. The subject is instructed on appropriate joint motion trajectories and muscle contraction via robot assistance and FES.

**Figure 3:**
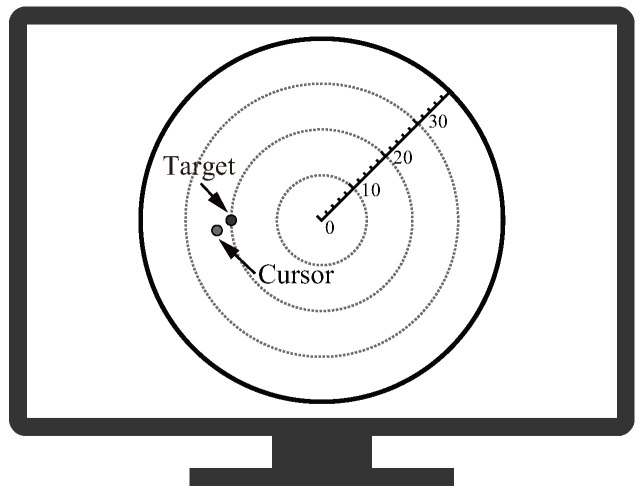
Example of visual feedback.

The training mode with visual feedback involves voluntary subject movement with arbitrary timing to reach a target point within $T_{\mathrm{train}}$ s. During the period $T_{\mathrm{feedback}}$ s after reaching the target point, the subject feeds back the positions of the target point and the arrival point on the screen to support training with exercise self-evaluation (Figure 3).

#### 2.2.4. Stimulation Level Calculation

FES is used in this method to induce muscle contraction to produce the target angle determined by Equation (4) based on the estimated motion. It is known that there is a nonlinear relation between the current and the wrist joint angle [10]. In this study, the following equation is used as a model of the current–joint angle characteristics during electric stimulation:

(5) $$\begin{array}{c} \theta \left(t\right)=\frac{d}{1+\exp\{b(c-I(t))\}} \end{array}$$

where *t*, θ(t), and I(t) are the time, joint angle, and current, respectively, and *b*, *c*, and *d* are real variables. In addition, *b*, *c*, and *d* represent the magnitude of the fluctuation of the joint angle, rapidity of the rise, and maximum value of the joint angle, respectively. The joint angle can be controlled using the inverse function of Equation (6) via electrical stimulation based on

(6) $$\begin{array}{c} I\left(t\right)=c-\frac{1}{b}\log\left(\frac{d}{\theta (t)}-1\right). \end{array}$$

Each parameter is determined using the least squares method from joint angles with stepwise current increases. Based on the results, the value of the current I(t) is determined from the target angle θ(t) calculated using Equation (4) and open-loop control is performed.

**Figure 4:**
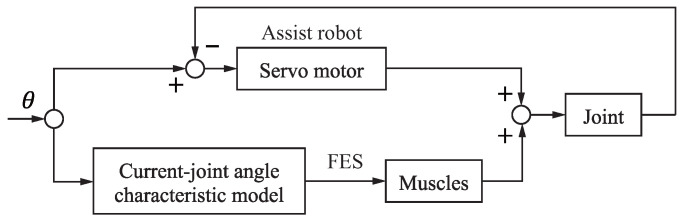
Control block.

Figure 4 shows the assistance robot and the FES, the latter of which controls joint motion by applying I(t) to the main active muscle based on θ(t) while simultaneously performing closed-loop servomotor control. As this control does not fully take into account the dynamic characteristics of the musculoskeletal system in response to electrical stimulation, there may be some conflict with the robot control angle. However, the FES-generated joint torque is much smaller than that of the assistance robot, meaning that the robot control dominates motion trajectory instruction. Future work will require closed-loop FES control with application to both the main active muscle and antagonist muscles to reduce competition with robot assistance.

## 3. Pointing Evaluation

Quantitative evaluation was applied to determine motion accuracy, stability, and smoothness, in addition to the conventional endpoint error index [16]. Here, *N* is the data set for *T* seconds at a sampling frequency of $f_{s}$ for the coordinate values in motion $P_{t}\in {ℜ}^{2}$.

- Endpoint error: *e*
- Maximum lateral deviation: $e_{⊥,\max}$
- Endpoint standard deviation: *s*
- Orbit correlation coefficient: $r_{\mathrm{orbit}}$
- Jerk cost: $j_{c}$
- Normalized mean velocity: ${\bar{v}}_{\mathrm{norm}}$

The endpoint error *e* and maximum lateral deviation $e_{⊥,\max}$ are used to evaluate the movement accuracy, representing the degree of matching with the specified motion. Smaller values indicate greater precision. The endpoint error *e* is calculated as follows, where $P_{t}$ represents the target-point coordinates:

(7) $$\begin{array}{c} e=\left|P_{t}-P\left(T\right)\right|. \end{array}$$

The coordinate values orthogonal to the direction of movement are denoted by $P_{⊥}\left(t\right)$, and the maximum lateral deviation $e_{⊥,\max}$ is determined by

(8) $$\begin{array}{c} e_{⊥,\max}=\max\left\{P_{⊥}\left(t\right)\right\}. \end{array}$$

The endpoint standard deviation *s* and the orbit correlation coefficient $r_{\mathrm{orbit}}$ are utilized as indicators of movement stability, with smaller and larger values respectively indicating higher movement reproducibility and stability; *s* is based on the number of trials *M*, and the parallel component of the coordinate value to the movement direction $P_{\|}\left(t\right)$ is calculated as follows:

(9) $$\begin{array}{c} e\left(i\right)={\left(P_{i,⊥}\left(T\right)-\bar{P_{⊥}\left(T\right)}\right)}^{2}+{\left(P_{i,\|}\left(T\right)-\bar{P_{\|}\left(T\right)}\right)}^{2}, \end{array}$$

(10) $$\begin{array}{c} s=\sqrt{\frac{1}{M}\sum_{i}^{M}e\left(i\right)}. \end{array}$$

The orbit correlation coefficient $r_{\mathrm{orbit}}$ is calculated using the correlation coefficient between the sequences x,y (denoted as r(x,y)), using

(11) $$\begin{array}{c} r_{\mathrm{orbit}}=\frac{1}{M(M-1)}\sum_{i=1}^{M}\sum_{j=1,j\neq i}^{M}r\left(P_{i,\|},P_{j,l}\right). \end{array}$$

The jerk cost $j_{c}$ represents the total rate of change in movement acceleration, with lower values indicating smoother motion. The normalized mean velocity ${\bar{v}}_{\mathrm{norm}}$ reflects the sharpness of the movement velocity peaks, with higher values representing smoother motion [22]. Accordingly, these indices were adopted to evaluate smoothness in pointing tasks. The jerk cost $j_{c}$ was calculated via

(12) $$\begin{array}{c} j_{c}=\frac{1}{2}\int_{0}^{T}{\left(\frac{d^{3}P_{\|}\left(t\right)}{dt^{3}}\right)}^{2}dt. \end{array}$$

The normalized average velocity ${\bar{v}}_{\mathrm{norm}}$ and velocity *v* were calculated as

(13) $$\begin{array}{c} v_{\|}\left(t\right)=\frac{dP_{\|}\left(t\right)}{dt},\;{\bar{v}}_{\mathrm{norm}\;}=\frac{\bar{v_{\|}}}{\max\left\{v_{\|}\right\}}. \end{array}$$

## 4. Experiment

### 4.1. Conditions

This experiment was conducted for pointing based on non-dominant-side wrist movements made by twelve non-paralyzed male subjects. Six of these (A–F) were instructed using the hybrid system, while the others (G–L) were trained via visual feedback.

An AMP04 electrode (Harada Electronics Industry, Sapporo, Japan)was used to monitor EMG signals, with application to four channels (L = 4) on the skin surface near the flexor carpi ulnaris, flexor carpi radialis, extensor carpi ulnaris, and extensor carpi radialis. The sampling frequency was set to $f_{s}=1000$ Hz, while the signal processing parameters were set to $f_{c}^{\mathrm{low}}=1$ Hz, $f_{c}^{\mathrm{high}}=250$ Hz, and $f_{c}=1$ Hz. The measurement system was grounded to reduce the effect of hum. SEN-8203 (Nihon Kohden Corp., Tokyo, Japan) and Isolator SS-104J (Nihon Kohden Corp.) electrical stimulators were used, and the proposed robot was used for training and evaluation. FES parameters were set to a frequency of 50 Hz and a duration of 0.2 ms. The stimulation electrodes were applied to the subjects by trial and error based on anatomical expertise at the positions of the target muscles to induce flexion, radial deviation, extension, and ulnar deviation. After this process, many of the electrodes were finally applied near the flexor carpi ulnaris, flexor carpi radialis, extensor carpi ulnaris, and extensor carpi radialis.

In R-LLGMN, the network architecture parameters were C=4, $K_{c}=2$, $M_{c,k}=2$, and T=50 and the component for each unit in the third layer was two. In the R-LLGMN learning process, the subjects performed each movement once and 0.5 s of data output (500 samples) were recorded. Thus, the dataset for all movements consisted of 2000 samples. The network was initialized and trained for each subject.

### 4.2. Method

**Figure 5:**
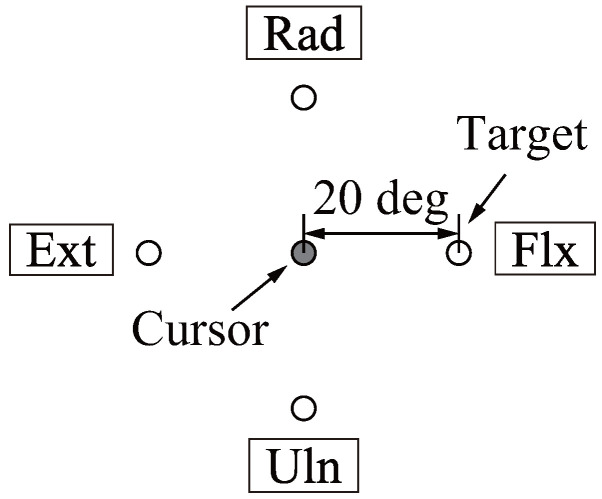
Training tasks.

Figure 5 details the training tasks. The subjects gripped the evaluation robot and performed pointing movements towards the target joint angles with flexion, radial deviation, extension, and ulnar deviation with a target joint angle of 20 degrees. The parameters were set as $T_{\mathrm{instruct}}=2$ s, $T_{\mathrm{train}}=3$ s, $T_{\mathrm{feedback}}=1$ s.

**Figure 6:**
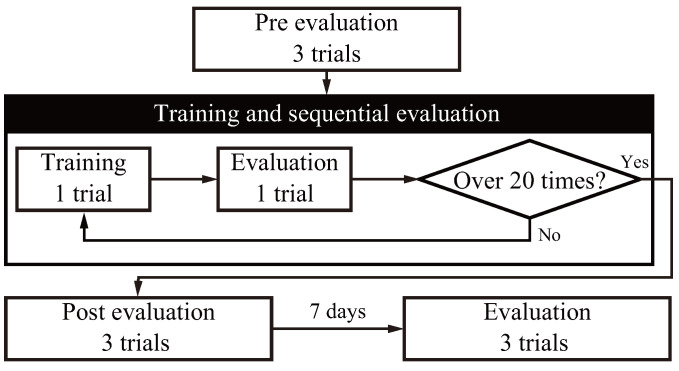
Experiment process.

In this experiment, training based on the hybrid system and visual feedback was conducted, and evaluation trials were conducted without instruction or feedback. Figure 6 shows the experimental flow. Both groups were pre-evaluated three times without feedback, then 20 sessions of training and sequential evaluation were conducted alternately for each method. Three post-evaluations were conducted after the training, and retention was evaluated seven days later. Pointing movements were made in four directions per trial, while Bonferroni multiple comparison testing was performed to evaluate inter- and intra-group differences across each evaluation stage for the computed metrics and to avoid Type I errors in inter-group comparison.

**Figure 7:**
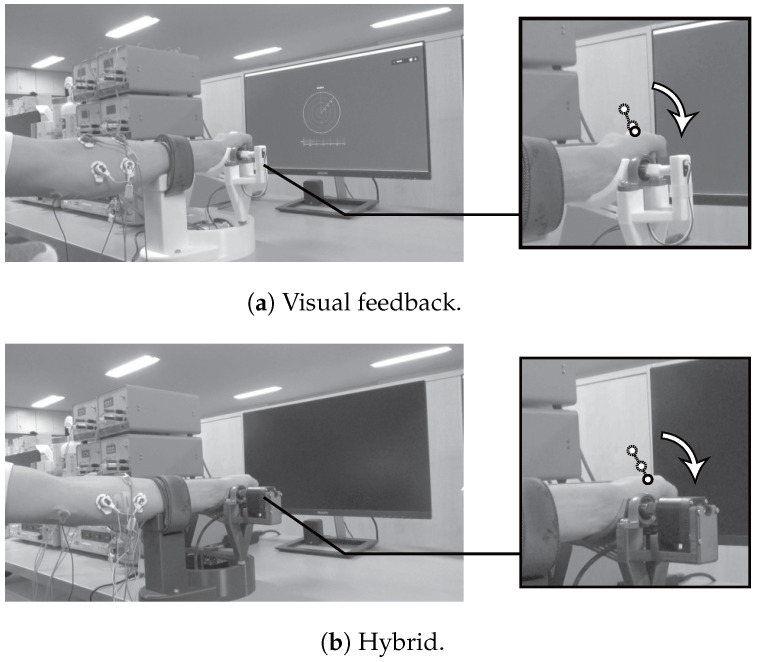
Training scenes. The arrows indicate the direction of movement, and the circles indicate the points of transition of the motion.

Figure 7 illustrates the training, in which on-screen visual feedback was provided and ideal instruction with the hybrid system was provided using assistance robotics and FES with movement determined from EMG signals. The subjects were asked to relax and to pay attention to the directions on movement and muscle contraction. The system was set to activate when a correct identification was made to equalize the number of training sessions for all subjects.

The experiment was conducted with the approval of the Ethics Committee of Fukuyama Memorial Hospital (Approval Number 20231001).

### 4.3. Results

**Figure 8:**
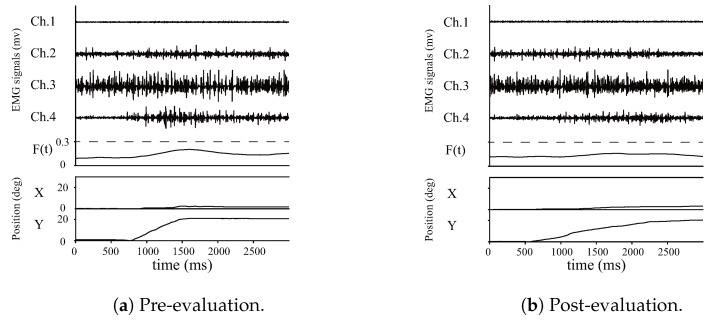
Examples of EMG signals, muscle activation level, and position.

Figure 8 shows an example of EMG signals, muscle activation level, and joint angle measurements for the ulnar flexion of Subject E during the evaluation trial. From the top, EMG signals, muscle activation level, and joint angles (X: flexion–extension direction; Y: radial deviation–ulnar deviation direction) are shown, with the horizontal axis indicating elapsed time from the start of joint movement. The joint angle results indicate slower change after training.

Correct identification was tied to the system drive in order to align the number of training sessions for all subjects. However, this made it difficult to quantitatively evaluate the classification performance of the system as a whole. Nevertheless, results from extensive previous research on R-LLGMN-based EMG signal classification have shown high identification ratios. For example, Bu et al. repeatedly identified signals for five subjects and reported an overall average identification ratio of approximately 93% [23]. Quantitative evaluation of identification performance with the proposed method is still required.

**Figure 9:**
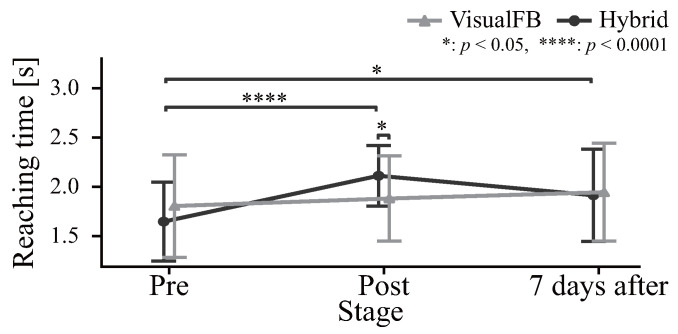
Reaching time.

Figure 9 shows the duration of movement for each group. The hybrid group showed a significant increase in the arrival time from pre- to post-training; however, no significant change was observed in the visual feedback group across the pre-training, post-training, and retention evaluations.

**Figure 10:**
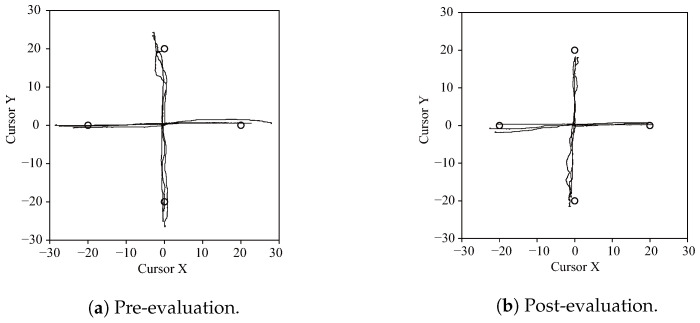
Pointing trajectories.

Figure 10 shows the pointing motion trajectories of subject I pre- and post-evaluation. The vertical axis represents the joint angle in the directions of radial deviation and ulnar deviation, while the horizontal axis represents the joint angle in the directions of flexion and extension. It can be seen that pointing is closer to the target point before training than after. Motion swing in the non-movement direction is also reduced, and motion is reproduced with a more linear path.

**Figure 11:**
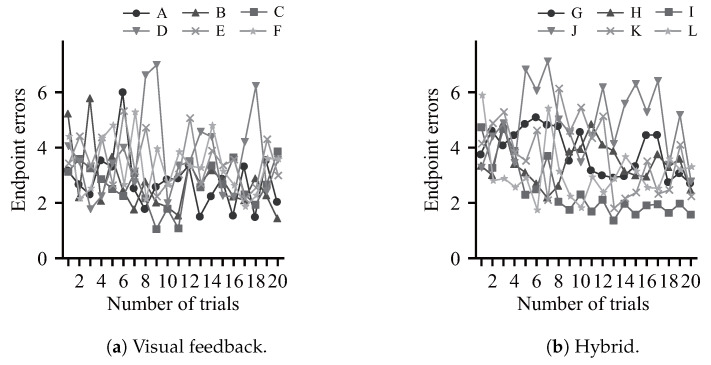
Endpoint errors for the sequential evaluation.

Figure 11 shows the sequential evaluation for the transition of pointing errors with each training condition. The vertical axis represents endpoint errors based on the norm between the target position and the final destination, while the horizontal axis represents the number of trials. The initial sequential evaluation scores were similar for both groups, though all subjects in the visual feedback group (A–F) showed lower endpoint errors *e* than most of the hybrid group (G, H, J, K), indicating that the errors decreased at an early stage. However, the errors tended to stagnate and then fluctuate in the second half of the training. All hybrid group subjects except J showed little error fluctuation and a decreasing trend in the last half of training after the fifteenth session.

**Figure 12:**
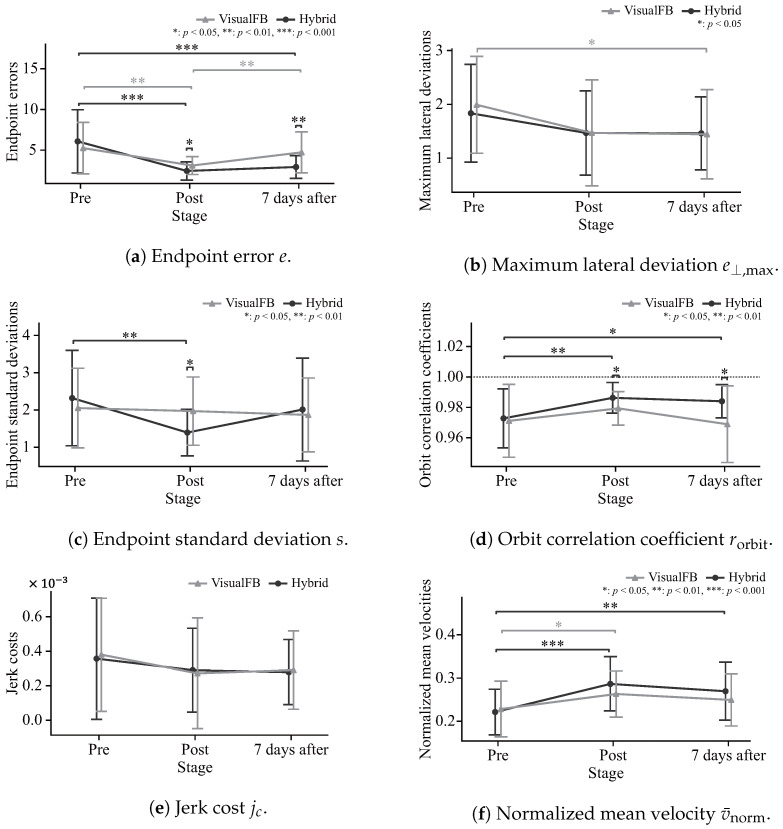
Evaluation indices.

Figure 12a shows the endpoint errors *e* for each group along with the scores in pre-, post-, and retention evaluation stages. Here, Bonferroni multiple comparison testing was performed to evaluate inter-group and intra-group differences across each evaluation stage for the computed metrics. There was a significant decrease in errors for both groups from pre- to post-evaluation as well as between the groups, with 5% significance in post-evaluation. These results correspond to pointing closer to the target indicated in Figure 10. In the retention evaluation after seven days, the visual feedback group showed a significant increase in the error, while the hybrid group did not. There was a considerable difference between the two groups at 1% significance.

Figure 12b shows the maximum lateral deviation $e_{⊥,\max}$ for each group, with values tending to decrease from pre- to post-evaluation for both groups. This is consistent with the reduction in motion blur and the realization of more linear motion, as shown in Figure 10. There was no change in retention evaluation after seven days in either group.

Figure 12c shows the endpoint standard deviation *s* for each group. The visual feedback group did not change from pre- to post-evaluation, but the hybrid group showed a considerable decrease at 1% significance. In the retention evaluation, the visual feedback group remained at the same level and the hybrid group showed an increasing trend, with both showing a slight decrease from pre-evaluation.

In Figure 12d, both groups exhibit an increased orbit correlation coefficient $r_{\mathrm{orbit}}$ from pre-evaluation to post-evaluation, with a considerable difference in the hybrid group at 1% significance. Post-evaluation exhibits a significant difference between the two groups at the 5% level. From post-evaluation to retention evaluation, the hybrid group shows a gradual decreasing trend and the visual feedback group shows a greater one. The retention score shows a considerable difference between the two groups at 1% significance.

In Figure 12e, both groups show a gradual decrease in thhe jerk cost $j_{c}$ from pre- to post-evaluation. There is no change in either group from post-evaluation to retention evaluation.

In Figure 12f, the normalized mean velocity ${\bar{v}}_{\mathrm{norm}}$ for both groups increases from pre- to post-evaluation, with 5% significance for the visual feedback group and 0.1% for the hybrid group. From post-evaluation to retention evaluation, both groups show a decreasing trend.

Table 1 compares the improvement ratios before and after training for each group. The values were normalized based on the pretraining percentages, and Mann–Whitney U tests were performed to evaluate the significance of the inter-group differences. It can be seen that both groups showed improvement for all indices. The improvement ratio of the hybrid group was high for certain indicators (endpoint errors *e*, endpoint standard deviation *s*, orbit correlation coefficient $r_{\mathrm{orbit}}$, normalized mean velocity ${\bar{v}}_{\mathrm{norm}}$) where significant amelioration was observed.

### 4.4. Discussion

In Figure 9, the subject arrival times in the hybrid group coincide with the instructed motion trajectory $T_{\mathrm{instruct}}$ at 2 s, indicating learning and reproduction of assisted motion. Conversely, the visual feedback group only receives arrival point information. Accordingly, it can be assumed that no change in movement time occurred.

Figure 11 shows that the subjects in the visual feedback group showed an early reduction in error compared to the hybrid group. However, the reduction in error tended to fluctuate from the middle of the training period. Subjects were provided with extrinsic visual feedback indicating the results of the exercise based on the Knowledge of Results (KR) approach. In motor learning, KR values with high frequency and accuracy reduce awareness of intrinsic feedback from human proprioceptive senses and inhibit motor learning [24]. The subjects in the visual feedback group may not have learned significantly after a certain level of error reduction due to feedback reliance. Conversely, the hybrid system consistently advised a fixed trajectory, allowing learning with little error fluctuation and no significant change in motion during the process of training repetition. This suggests that the training methods have different characteristics, and that more effective motor learning may be achieved via a combination of training and instruction.

Figure 12a,b shows a significant decrease in the endpoint error *e* and a decreasing trend in the maximum lateral deviation $e_{⊥,\max}$ after training, indicating that pointing movement accuracy was improved via training with the proposed method. The reduced endpoint standard deviation *s* (Figure 12c) and increased orbit correlation coefficient $r_{\mathrm{orbit}}$ (Figure 12d) indicate that movement reproducibility was improved and stable pointing was achieved. The decreasing trend in the jerk cost $j_{c}$ (Figure 12e) and the increased normalized mean velocity ${\bar{v}}_{\mathrm{norm}}$ (Figure 12f) indicate smoother motion, implying improved feedforward control capability based on pointing position prediction. This suggests that instruction with the hybrid system supports motor learning equivalent to active training in motion accuracy, stability, and smoothness.

Significant differences between the hybrid group and the visual feedback group were shown for the endpoint error *e* and the orbit correlation coefficient $r_{\mathrm{orbit}}$ in the retention evaluation (Figure 12a,d), suggesting that instruction with the hybrid system provides greater post-training retention. The proposed method involves the assumption that consistent movement guidance via trajectory and muscle contraction instruction from assistance robotics together with FES based on movement estimation contribute to movement retention.

Figure 12c shows that improvement in the endpoint standard deviation *s* from pre- to post-evaluation tended to differ between the groups. Table 1 highlights differences in the degree of improvement by index. This suggests differences in motor learning effects between training with visual feedback and the proposed method. It can be considered that more effective training may be possible by adapting the method to the motor characteristics of the subjects in the study. However, the differences in the method and content of information presentation between the groups in the experiment must be considered, and further research is needed to determine effects from the different learning effects.

While there were more subjects here than in previous research, the sample size may still be insufficient for more detailed evaluation of the proposed method. Therefore, a larger cohort and statistical evaluation of the method’s motor learning effectiveness remain necessary.

Users with upper limb paralysis often have difficulty learning joint movement based on trial and error. However, by applying the proposed system it is possible to teach movement via robot assistance and FES, which is expected to enable rehabilitation effects similar to those of motor learning with the non-paralyzed subjects in this paper. Meanwhile, the difficulty of evaluating EMG signals in relation to paralysis and intended movement must be noted. Further verification for the determination of motion intent from EMG signals in paralysis and the applicability of the system (e.g., joint control with assistive robotics for spasticity and contractures) is warranted.

## 5. Conclusions

This paper presents a detailed study of motor learning effects on pointing movements using EMG-driven hybrid rehabilitation based on motion evaluation from a probabilistic neural network. In the experiment, six participants in the hybrid group and six participants in the visual feedback group were trained to perform pointing movements with the wrist joint of the non-dominant hand. The results showed that the hybrid system was as effective as the training with visual feedback, and a retention effect after the training was observed. Passive instruction with the proposed system is expected to be effective in promoting motor learning and restoring motor function in paralyzed patients who have difficulty in performing voluntary movements. Future clinical application will require quantitative observation of effects from motor and sensory function reconstruction. For example, the results before and after intervention should be evaluated using clinical scores such as FMA and MAS, and the applicability of the proposed method should be discussed with therapists.

More research will be conducted to evaluate the proposed method in clinical trials with verification of related rehabilitation. Training with passive instruction and active feedback will also be developed to further evaluate related motor learning effects.

## Figures and Tables

**Table 1:** Comparison of improvement ratios for each index (post-training).

Index	Visual Feedback [%]		Hybrid (Proposed) [%]		*p*
	Mean	SD	Mean	SD	
Endpoint error *e*	40.86	21.02	59.88	18.42	<0.001 ***
Maximum lateral deviation $e_{⊥,\max}$	26.20	49.42	19.95	42.66	0.519
Endpoint standard deviation *s*	3.96	44.61	39.87	26.98	0.006 **
Orbit correlation coefficient $r_{\mathrm{orbit}}$	0.85	1.13	1.39	1.04	0.051
Jerk cost $J_{c}$	28.31	84.61	18.66	68.23	0.136
Normalized mean velocity ${\bar{v}}_{\mathrm{norm}}$	15.22	23.34	29.38	28.21	0.093

Note: *** p<0.001, ** p<0.01.

## Data Availability

The data presented in this study are available on request from the corresponding author.
